# Liposome-Mediated Delivery Improves the Efficacy of Lisosan G against Retinopathy in Diabetic Mice

**DOI:** 10.3390/cells12202448

**Published:** 2023-10-13

**Authors:** Rosario Amato, Alberto Melecchi, Laura Pucci, Alessio Canovai, Silvia Marracci, Maurizio Cammalleri, Massimo Dal Monte, Carla Caddeo, Giovanni Casini

**Affiliations:** 1 Department of Biology, University of Pisa, 56126 Pisa, Italy; rosario.amato@unipi.it (R.A.); a.melecchi@student.unisi.it (A.M.); a.canovai@student.unisi.it (A.C.); silvia.marracci@unipi.it (S.M.); maurizio.cammalleri@unipi.it (M.C.); massimo.dalmonte@unipi.it (M.D.M.); 2 Institute of Agricultural Biology and Biotechnology, National Research Council (CNR), 56124 Pisa, Italy; pucci@ibba.cnr.it; 3 Interdepartmental Research Center Nutrafood “Nutraceuticals and Food for Health”, University of Pisa, 56124 Pisa, Italy; 4 Department of Life and Environmental Sciences, University of Cagliari, 09042 Cagliari, Italy

**Keywords:** nutraceutical, bioavailability, nanocarriers, diabetic retinopathy, electroretinography, oxidative stress, inflammation, blood–retinal barrier

## Abstract

Nutraceuticals are natural substances whose anti-oxidant and anti-inflammatory properties may be used to treat retinal pathologies. Their efficacy is limited by poor bioavailability, which could be improved using nanocarriers. Lisosan G (LG), a fermented powder from whole grains, protects the retina from diabetic retinopathy (DR)-induced damage. For this study, we tested whether the encapsulation of LG in liposomes (LipoLG) may increase its protective effects. Diabetes was induced in mice via streptozotocin administration, and the mice were allowed to freely drink water or a water dispersion of two different doses of LG or of LipoLG. Electroretinographic recordings after 6 weeks showed that only the highest dose of LG could partially protect the retina from diabetes-induced functional deficits, while both doses of LipoLG were effective. An evaluation of molecular markers of oxidative stress, inflammation, apoptosis, vascular endothelial growth factor, and the blood–retinal barrier confirmed that the highest dose of LG only partially protected the retina from DR-induced changes, while virtually complete prevention was obtained with either dose of LipoLG. These data indicate that the efficacy of LG in contrasting DR is greatly enhanced by its encapsulation in liposomes and may lay the ground for new dietary supplements with improved therapeutic effects against DR.

## 1. Introduction

A large variety of substances of natural origin have attracted interest for their therapeutic potential in different retinal pathologies, and a considerable amount of both experimental and clinical data have been collected and extensively reviewed in recent years [1,2,3,4,5,6,7,8,9]. Nutraceuticals are characterized by their important anti-oxidant capacity, acting either as inducers of anti-oxidant enzyme expression or as scavengers of reactive oxygen species [10], and by their anti-inflammatory properties [11]. They can be used as natural dietary supplements and are unlikely to induce collateral side effects [12].

Diabetic retinopathy (DR) is characterized by oxidative stress deriving from high glucose-induced biochemical alterations and leading to inflammation, excitotoxicity, and neurotrophin depletion, which ultimately promote neurodegenerative phenomena characterizing the early stages of the disease and likely preceding vascular abnormalities [13,14,15]. Among the nutraceuticals tested for their potential therapeutic use in DR [1,5,7], Lisosan G (LG) deserves attention due to its powerful anti-oxidant and anti-inflammatory capacities. This compound is a fermented powder obtained from whole grains and is rich in bioactive substances, including both flavonoid and non-flavonoid polyphenols, alpha-lipoic acid, polyunsaturated fatty acids, and vitamins, among others [16,17,18,19]. We have shown that, in retinas of rats with streptozotocin (STZ)-induced diabetes, oral LG administration via gavage restored electroretinogram (ERG) responses; reduced retinal cell apoptosis, inflammation, vascular endothelial growth factor (VEGF) expression and VEGF receptor activation; and prevented damage to the blood–retinal barrier (BRB) [20]. Interestingly, LG was also found to protect the eyes of *Drosophila melanogaster* from oxidative stress and neurodegeneration induced by a high-sugar diet [21].

In an investigation on the possible protective effects of LG against retinal damage induced by glaucoma, we found that glaucoma progression was significantly reduced in mice that had free access to an aqueous LG solution in place of drinking water for two months. These observations showed that LG can effectively provide anti-oxidant efficacy following its spontaneous assumption by animals without provoking detectable side effects over extended periods of time. In this context, we also demonstrated that four of the main LG components (nicotinamide, gallic acid, 4-hydroxybenzoic acid, and quercetin) could be detected in mouse retinas after LG administration by gavage, although only a limited fraction of their initial dosage was found within the target tissue, and the permanence of detectable amounts of these compounds was in the order of minutes [22]. This evidence suggests that the potential efficacy of LG might be significantly reduced by limited access at the target organ.

In general, an important limiting factor for the use of nutraceuticals is their poor bioavailability, a pharmacokinetic term referring to the fraction of bioactive compounds that are circulated in the blood without undergoing alterations [23]. Indeed, ingested foods of vegetal origin are modified in their structure/properties by digestive processes and hepatic metabolism so that only a small fraction of the ingested quantity reaches the target tissues. This limitation has important consequences for the effective use of nutraceuticals, and new methods to improve nutraceutical bioavailability are being exploited through the use of nanoparticles [24]. Among nanocarriers, liposomes represent a reliable system for the delivery of bioactive substances due to their unique composition and structure, the latter of which is similar to those of biological membranes, making them highly biocompatible and capable of carrying either hydrophilic or lipophilic compounds [25,26]. We have recently demonstrated that LG could be effectively encapsulated into Eudragit-liposomes with a good entrapment efficiency and storage stability over three months, providing the preservation of LG anti-oxidant properties [27]. In the present work, we tested whether LG-encapsulated liposomes (LipoLG) could improve the protective effects of LG against DR in a mouse model of type I diabetes.

## 2. Materials and Methods

### 2.1. Reagents

LG powder was provided by Agrisan Company (Larciano, Pistoia, Italy). The liposome components Soy lecithin and Eudragit^®^ L100 were purchased from Evonik Industries AG (Essen, Germany). Streptozotocin (STZ; cat. S0130) was purchased from Sigma Aldrich-Merck (St. Louis, MO, USA). RIPA lysis buffer (cat. sc-24948A) was obtained from Santa Cruz Biotechnology (Dallas, TX, USA), while the phosphatase and proteinase inhibitor cocktails were obtained from Roche Applied Science (Indianapolis, IN, USA). Micro BCA Protein Assay (cat. 23235) and β-mercaptoethanol (cat. J66742) was purchased from Thermo Fisher Scientific (Waltham, MA, USA). SDS (cat. A02263) and non-fat milk (cat. A0830) powders were obtained from PanReac AppliChem (Milano, Italy). Clarity western enhanced chemiluminescence substrate (cat. 1705061) was purchased from Bio-Rad Laboratories, Inc. (Hercules, CA, USA). The specifics of the primary and secondary antibodies used are reported in Table 1.

### 2.2. Animals

All the procedures were performed in compliance with the ARVO Statement for the Use of Animals in Ophthalmic and Vision Research, the EU Directive (2010/63/EU), and the Italian guidelines for animal care (DL 26/14; Italian Ministry of Health, permission number 292/2022-PR). A total of 46 C57BL/6J mice of both sexes (ENVIGO, San Pietro al Natisone, Udine, Italy) were used in these studies. They were kept in a regulated environment (23 ± 1 °C, 50 ± 5% humidity) with a 12 h light/dark cycle (lights on at 8:00 a.m.) with food and water ad libitum. As described below, for different groups of mice, water was replaced by LG, LipoLG, or empty liposomes (i.e., liposomes without LG; Lipo).

### 2.3. Diabetes Induction

Diabetes was induced in 5 week-old animals by means of a daily intraperitoneal injection of STZ (50 μg/g body weight) dissolved in sodium citrate buffer (137 mM, pH 4.5) for 5 consecutive days [28]. Control mice (*n* = 4) received equivalent volumes of the citrate buffer solution. Three days after the last injection, the mice were tested for blood glucose levels using a OneTouch Ultra glucometer (LifeScan Inc., Milpitas, CA, USA). Those showing a stable glycemia ≥ 250 mg/dL were considered diabetics [28] and were randomly assigned to the different experimental groups (Table 2). The mice were followed-up for glycemia and weight every week.

### 2.4. Preparation of LG Extract and LipoLG

LG has been registered by the Italian Ministry of Health as a nutritional supplement. It is a powder that can be obtained via the fermentation and drying of whole wheat flour from *Triticum aestivum* grains, as described previously [20,22]. An aqueous extract of LG was obtained by dispersing LG powder in water (83 mg/mL), followed by sonication and centrifugation at 2300× *g* for 10 min at 4 °C. Then, the supernatant was collected and stored in the dark at 4 °C until use (stock solution). LipoLG were prepared as described recently [27]. Briefly, soy lecithin and Eudragit^®^ L100 (a gastro-resistant polymer) were weighed and dispersed in a 1:1 LG stock solution:water blend. LipoLG were small in size (mean diameter around 130 nm) and characterized by good homogeneity (PI < 0.3) and a negative surface charge (around −30 mV). An analysis of phenolic compounds showed that LipoLG had a phenolic content comparable to that of the LG stock solution, which was reflected in comparable anti-oxidant power, indicating that the encapsulation process preserved the LG components and their bioactivity [27]. Lipo were prepared by replacing the LG stock solution with an equal volume of water.

### 2.5. LG and LipoLG Administration

**Figure 1:**
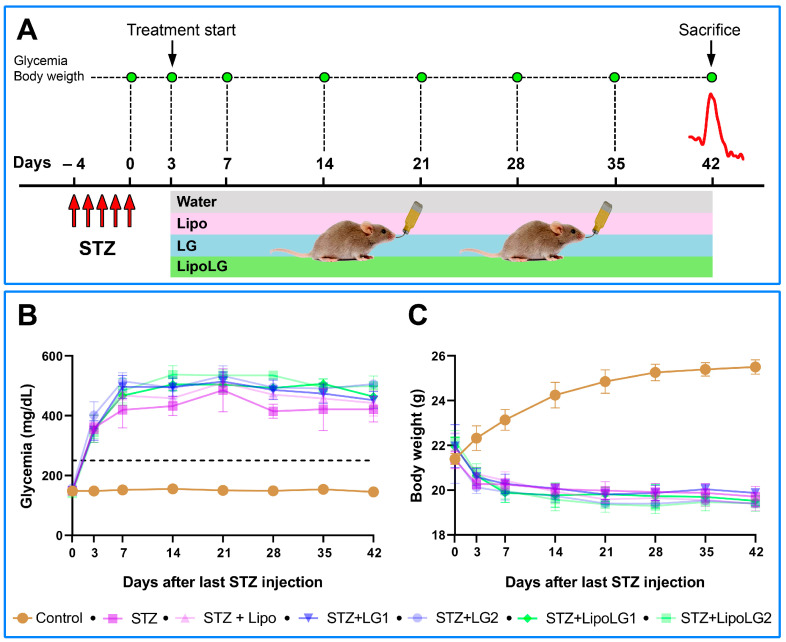
(**A**) Treatment/testing paradigm of the study. The mice received a daily STZ i.p. injection for five consecutive days. Three days after the last STZ injection, glycemic levels were measured; the animals were assigned to different experimental groups, and the protocol of Lipo, LG, or LipoLG administration begun. ERG recordings (red line) were performed after 6 weeks from the last STZ injection. After the ERG recordings, the mice were sacrificed and their retinas were used for Western blot analyses. The glycemia and the body weight of the mice during the experimental period are shown in (**B**,**C**), respectively. The dashed line in (**B**) indicates glycemia = 250 mg/dL. *n* = 4 in all graphs.

The treatments with LG or LipoLG started three days after the last STZ injection (Figure 1A). The diabetic mice were divided into six groups (7 mice in each group). One group (STZ) had access to water, while for the other groups, water was replaced by Lipo diluted 1:10 (STZ + Lipo), a LG solution at 1:10 or 1:100 dilution (STZ + LG1 and STZ + LG2, respectively), or LipoLG at 1:10 or 1:100 dilution (STZ + LipoLG1 and STZ + LipoLG2, respectively). The animals that received citrate buffer instead of the STZ injections remained untreated and served as controls (*n* = 7). A description of the experimental groups is provided in Table 2. Preliminary observations revealed that the mice seemed to appreciate the taste of LG, and that the volume of LG solution consumed by the mice was similar to that of drinking water. The same was observed for Lipo and for LipoLG. As noted previously [22], a mouse drinks, on average, about 3 mL/day. Assuming an average mouse body weight of 20 g, the amount of LG delivered to each mouse with the 1:10 dilution of both LG and LipoLG corresponded to 1 g LG/Kg/day. Considering that, with the progression of diabetes, mice considerably increase their consumption of liquids, this amount was destined to increase. To avoid possible differences in the LG or LipoLG dosage in the diabetic mice drinking different volumes, both LG and LipoLG were given to the mice at a fixed volume of 10 mL/day, after which LG and Lipo LG were replaced by water. At the end of the experimental period, the numerosity within the different groups was decreased by one, two, or three units due to the mice experiencing health problems caused by diabetes (humane endpoint). To maintain homogeneity among group numerosity, four mice from each group were used for ERG recordings, and three of them were subsequently used for Western blot analyses.

### 2.6. Electroretinography

Electroretinographic recordings were performed six weeks after the last STZ injection (Figure 1A). The mice were subjected to a recording routine that included an analysis of the rod pathway with scotopic ERG (scERG), an analysis of the cone pathway with photopic ERG (phERG), and an analysis of retinal ganglion cell (RGC) activity with pattern-ERG (PERG). After overnight dark adaption, the mice were anesthetized via an intraperitoneal injection of Avertin (1.25% avertin/g body weight) and gently restrained in a custom-made stereotaxic apparatus, allowing for an unobstructed visual field. Body temperature was maintained at 37 °C using a feedback-controlled heating pad, and corneal moisture was maintained via the use of saline. The recording electrodes (0.2 mm loop-shaped silver wires) were carefully laid on the corneal surface of each eye using micro manipulators. Stainless steel needles were used as reference electrodes (one for each eye) and ground electrodes and inserted subcutaneously on the mouse scalp and at the tail root, respectively. In each recording session, scERG was performed first using a commercially available ERG setup (Retimax Advanced, CSO, Firenze, Italy), and a 10 cd⋅s/m^2^ flash stimulus was delivered via a Ganzfeld bowl. Five consecutive signals recorded simultaneously from each eye were averaged to reduce noise after amplification (5000-fold) and subsequently band-pass filtered (1–100 Hz). In the deriving waveform, the amplitude of the a-wave (baseline to trough) and that of the b-wave (trough to peak) were measured. Oscillatory potentials (OPs) were isolated from each endpoint scERG recording via band-pass filtering at 65–300 Hz to exclude interference from the a- and b-wave. The amplitudes of the main OPs (OP1, OP2 and OP3) were measured from the corresponding peaks to the preceding troughs, according to the ISCEV guidelines [29].

Immediately after the scERG session, the mice were light-adapted by exposing them to 40.0 cd⋅s/m^2^, rod saturation, and a green background light, delivered with a Ganzfeld bowl, for one minute. Thereafter, cone pathway stimulation was obtained by delivering 5 cd⋅s/m^2^ flashlight stimuli on the same adaptation background. The phERG responses simultaneously recorded from both eyes were amplified (5000-fold) and band-pass filtered (1–100 Hz). The waveform deriving from the average of 50 consecutive responses was analyzed for the b-wave (baseline to peak) and the photopic negative response (baseline to trough).

The phERG session was followed by PERG recordings. Using the same electrode setup, a visual stimulus consisting of black and white (98% contrast) bars contrast-reversing at 1 Hz temporal frequency and 0.05 cyc/deg spatial frequency was administered with a 19” light emitting diode display (area: 74° × 62°) aligned to the mouse cornea and spaced 25 cm from the mouse eye. Each pattern reversal-deriving signal was amplified (10,000-fold) and band-pass filtered (1–30 Hz). Overall, the responses deriving from 900 consecutive pattern reversals were averaged to reduce noise contamination by a factor of √900 = 30. PERG responses were analyzed by detecting the early positive inflection point (N35), the positive peak (P50), and the belated negative trough (N95) in each PERG waveform. Given the proportional variation of the positive (N35-P50) and negative (P50-N95) components of the PERG response, the P50-N95 amplitude (hereinafter referred to as PERG amplitude) was chosen as the main analytical parameter for the PERG analysis in order to optimize both the dynamic range of measures and the signal-to-noise ratio. The implicit time of the PERG response was also analyzed as time-to- P50 (hereinafter referred to as PERG latency).

### 2.7. Western Blotting

After the last ERG recording, the mice were sacrificed via cervical dislocation. The eyes were then enucleated, and the retinas were rapidly dissected by removing the cornea, sclera, and lens under a stereomicroscope. For our Western blotting assay, retinal samples (two retinas in each sample) were lysed via three sonication cycles in RIPA lysis buffer supplemented with phosphatase and proteinase inhibitor cocktails. The protein content was quantified by using the Micro BCA Protein Assay. Thirty micrograms of protein of each sample were mixed with 5X Laemmli sample buffer containing 20% SDS and 5% β-mercaptoethanol as reducing agents and then incubated at 95 °C for 5 min. The samples were separated by SDS-PAGE (4–20%; Bio-Rad Laboratories) and transferred onto nitrocellulose membranes (Bio-Rad). The membranes were blocked with 5% non-fat milk for 1h at room temperature and subsequently incubated overnight at 4 °C with a primary antibody (Table 1). After washing in 1X tris-buffered saline containing 0.05% Tween-20, the membranes were incubated for 1h at room temperature with a HRP-conjugated secondary anti-mouse or anti-rabbit antibodies (Table 1). The blots were developed by the Clarity western-enhanced chemiluminescence substrate, and the signal was detected using ChemiDoc XRS+ (Bio-Rad). The optical density (OD) of the target bands was evaluated using ImageLab 3.0 software (Bio-Rad). The data were normalized to the corresponding OD of β-actin or to the p65 subunit of nuclear factor kappa-light-chain-enhancer of activated B cells (NF-kB p65) as an appropriate loading control.

### 2.8. Statistics

All variables were tested using the Shapiro–Wilk test to certify their normal distribution. Hence, data were analyzed using two-way analysis of variance (ANOVA) followed by Tukey’s post hoc test. The results are expressed as mean ± SEM of the indicated *n* values (Prism 8; GraphPad software, San Diego, CA, USA). Differences with *p* < 0.05 were considered significant.

## 3. Results

### 3.1. Glycemia and Body Weight

Normal blood glucose levels ranged between 132 and 170 mg/dL during the six weeks of the experiment. These values were significantly higher in STZ mice, treated or not with Lipo, LG, or LipoLG, starting three days after the last STZ injection (Figure 1B). The body weight of the control mice ranged between 20.15 g and 26.87 g, and it was consistently lower in STZ mice, treated or not with Lipo, LG, or LipoLG, starting three days after the last STZ injection (Figure 1C). These results show early diabetogenic effects of STZ, such as prolonged hyperglycemia and weight loss, and the absence of major metabolic effects induced by Lipo, by LG, or by LipoLG.

### 3.2. Effects of LG and LipoLG on Retinal Function

The electroretinographic routine used in these studies was designed to obtain a comprehensive evaluation of the retinal function over time including dark- and light-adapted photoreceptoral, postreceptoral, and RGC activity.

#### 3.2.1. Scotopic ERG

**Figure 2:**
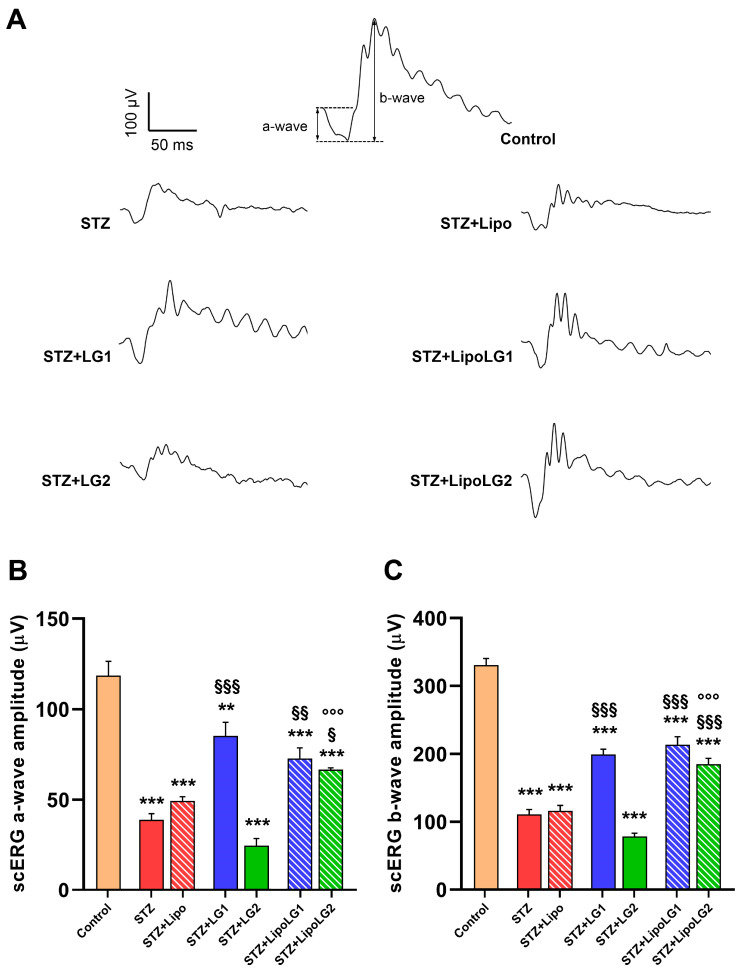
(**A**) Representative scERG recordings, with a- and b-waves shown in the control. (**B**,**C**) represent the amplitudes of the scERG a-wave and b-wave, respectively. ** *p* < 0.01, *** *p* < 0.001 vs. control; ^§^ *p* < 0.05, ^§§^ *p* < 0.01, ^§§§^ *p* < 0.001 vs. STZ; °°° *p* < 0.001 vs. STZ + LG2. *n* = 4 in all graphs.

As shown in the scERG representative traces of Figure 2A, there were evident differences in the recorded functional activities between mice receiving different treatments. In particular, the amplitude of the a-wave (related to the overall photoreceptoral activity, Figure 2B) was dramatically reduced to less than 50% in diabetic animals either untreated or treated with Lipo. The a-wave of diabetic animals treated with LG1 showed a significant recovery, similar to that of the animals treated with LipoLG1. In contrast, the scERG a-waves of diabetic mice treated with LG2 did not show any recovery and were similar to those of the animals in the STZ or STZ + Lipo groups, while those of the mice treated with LipoLG2 were similar to those recorded for mice treated with either LG1 or LipoLG1 and were significantly larger than those of the STZ + LG2 group. None of the treatments with LG or with LipoLG led to a full recovery since the a-wave amplitudes remained significantly lower than those of controls. A very similar pattern was observed for the b-wave (reflecting the overall post-receptoral response including bipolar cell and Müller cell activity, Figure 2C). In summary, these data indicated that an improvement in scERG responses (in both the a- and the b-wave) could be obtained in diabetic mice with LG1 but not with LG2, while both LipoLG1 and LipoLG2 were effective.

**Figure 3:**
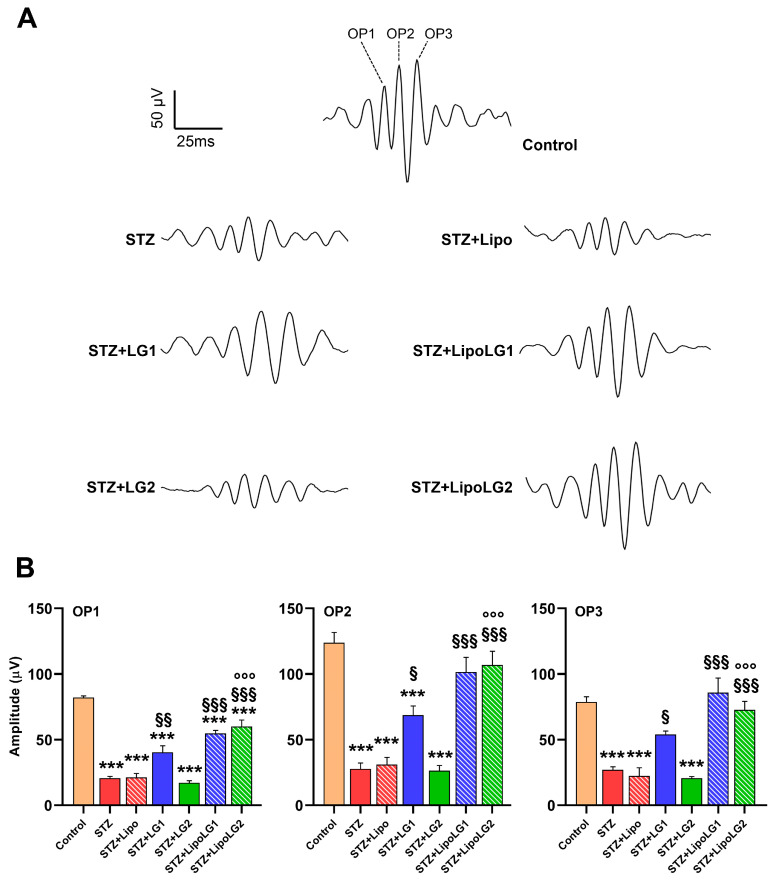
(**A**) Representative OPs recordings. (**B**) Amplitudes of OP1, OP2, and OP3 in the different experimental groups. *** *p* < 0.001 vs. control; ^§^ *p* < 0.05, ^§§^ *p* < 0.01, ^§§§^ *p* < 0.001 vs. STZ; °°° *p* < 0.001 vs. STZ + LG2. *n* = 4 in all graphs.

Consistent with the scERG data, the retrieval of OPs from the scERG responses (representative OPs are shown in Figure 3A) also revealed important differences among experimental groups. OPs are related to the activity of third-order neurons, including amacrine cells and RGCs, and they are composed of three main components: OP1, OP2, and OP3. As shown in Figure 3B, all three components were significantly reduced in the STZ and STZ + Lipo groups compared to the controls. The treatment with LG1 induced a significant recovery of the amplitudes of all three OPs, although those of OP1 and OP2 remained significantly lower than in the controls. Similarly, the treatment with LipoLG1 also induced significant increases in OP amplitudes and the complete recovery of OP2 and OP3, whereas the amplitudes in the STZ + LipoLG1 groups were not significantly different from those in the controls. Treatment with LG2 did not induce any effect in any of the OPs, as no significant differences were observed between the STZ and the STZ + LG2 groups. In contrast, treatment with LipoLG2 led to a significant increase in the OP1 amplitudes and the complete recovery of OP2 and OP3. The differences between the OP amplitudes in the STZ + LG2 and those in the STZ + LipoLG2 groups were highly significant. Overall, the data for the OP amplitudes showed important improvements induced by LG1 or LipoLG1 with similar efficacy. Most importantly, the treatment with LipoLG2 produced improvements similar to those obtained with LipoLG1, while LG2 was ineffective.

#### 3.2.2. Photopic ERG

**Figure 4:**
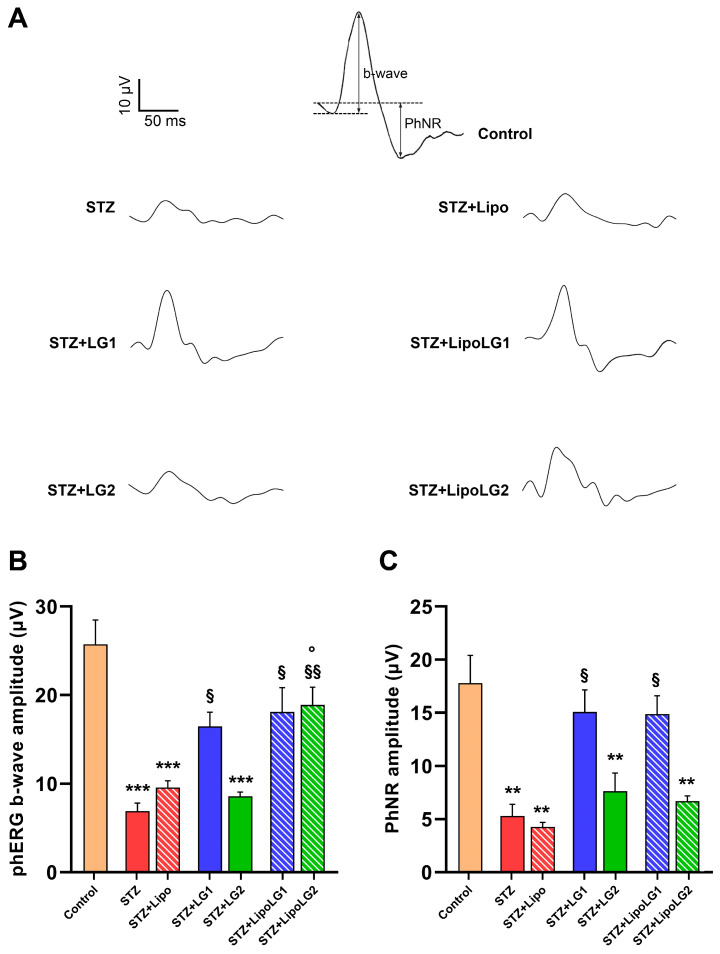
(**A**) Representative phERG recordings, with b-wave and PhNR shown in the control. (**B**,**C**) represent the amplitudes of the phERG b-wave and of the phNR, respectively. ** *p* < 0.01, *** *p* < 0.001 vs. control; ^§^ *p* < 0.05, ^§§^ *p* < 0.01 vs. STZ; ° *p* < 0.05 vs. STZ + LG2. *n* = 4 in all graphs.

Representative phERG responses are shown in Figure 4A. The amplitude of the phERG b-wave (Figure 4B) related to cone photoreceptors was dramatically reduced in the STZ and STZ + Lipo groups. Both treatments with LG1 and with LipoLG1 were effective in restoring phERG b-wave amplitudes similar to those of control mice. The phERG b-wave amplitude in the STZ + LG2 group was similar to that in STZ or in STZ + Lipo mice, indicating that the treatment with LG2 had no effect. In contrast, the treatment with LipoLG2 led to a response recovery similar to that recorded in the LG1- or LipoLG1-treated diabetic animals. Similar to the scERG findings, the values of the STZ + LipoLG2 group were significantly higher than those of the STZ + LG2 group. Regarding the PhNR amplitude (Figure 4C), which is generally related to RGC activity, it was significantly reduced in STZ and in STZ + Lipo mice compared to the controls. The treatments with LG1 or with LipoLG1 were equally efficient in rescuing phNRs similar to those of the control mice, while the lowest doses of both LG and LipoLG were not effective (Figure 4C).

Overall, the phERG data showed that the treatments with LipoLG conferred full protection, while those with LG did not, acting against the reduction in the phERG b-wave, while no differences between the LG and LipoLG treatments could be observed on the phNR.

#### 3.2.3. Pattern ERG

**Figure 5:**
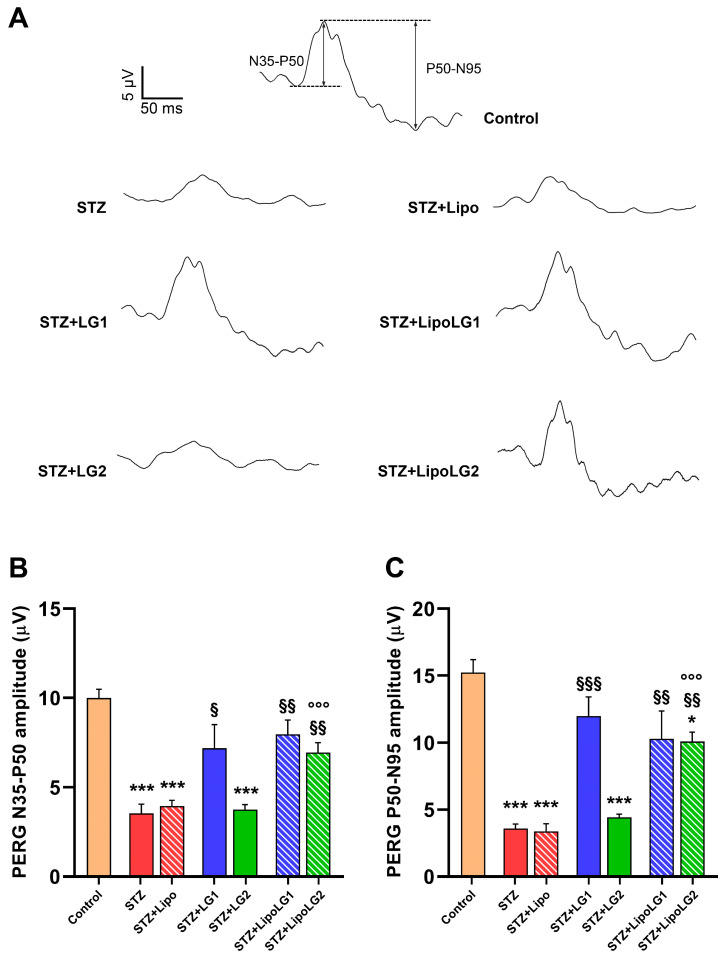
(**A**) Representative PERG recordings, with N35-P50 and P50-N95 waves shown in the control. (**B**,**C**) represent the amplitudes of the n35-p50 and of the p50-n95 waves, respectively. * *p* < 0.05, *** *p* < 0.001 vs. control; ^§^ *p* < 0.05, ^§§^ *p* < 0.01, ^§§§^ *p* < 0.001 vs. STZ; °°° *p* < 0.001 vs. STZ + LG2. *n* = 4 in all graphs.

PERG responses (representative traces are shown in Figure 5A) were recorded to investigate the functional activity of RGCs. Both the n35-p50 (Figure 5B) and the p50-n95 (Figure 5C) amplitudes were significantly decreased in the STZ and the STZ + Lipo groups, while complete recovery was observed in the retinas of mice treated with LG1, LipoLG1, or LipoLG2. The lowest dose of LG (LG2) was ineffective. Overall, the PERG data indicated an almost complete functional recovery of RGCs that could be obtained with either dose of LipoLG, while only LG1 (not LG2) was effective.

### 3.3. Effects of LG and LipoLG on Molecular Markers of DR

The Western blot analyses were performed after the ERG recordings. They were conducted to evaluate the levels of typical hallmarks of DR—oxidative stress, inflammation, glial reaction, apoptosis, VEGF expression, and BRB breakdown—in the different experimental groups.

**Figure 6:**
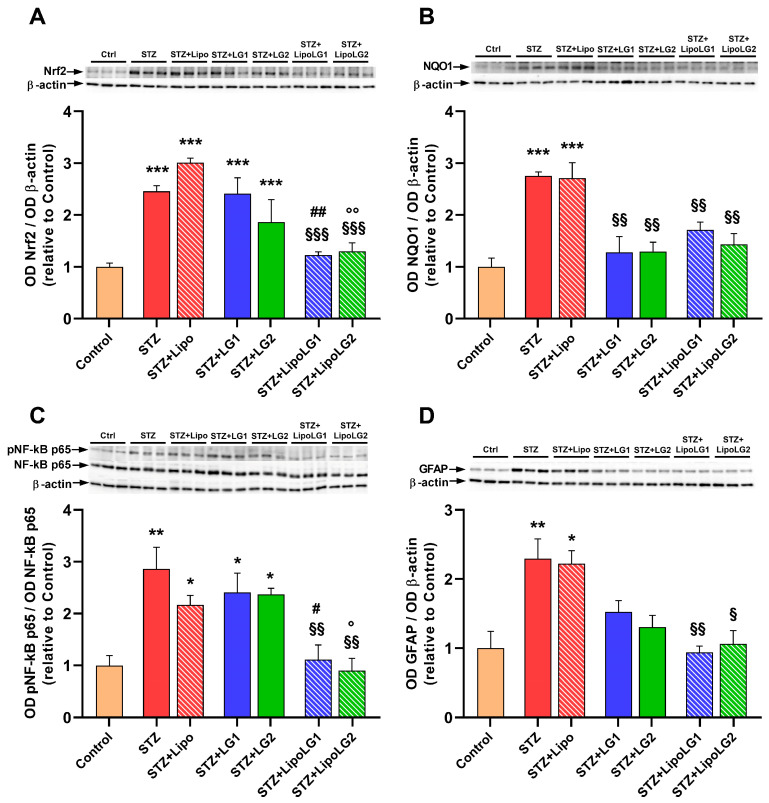
Western blot analysis showing representative immunoreactive bands and quantitative densitometric analysis of the protein levels of Nrf2 (**A**), NQO1 (**B**), the ratio pNF-kB p65/NF-kB p65 (**C**), and GFAP (**D**) in the different experimental groups. * *p* < 0.05, ** *p* < 0.01, *** *p* < 0.001 vs. control; ^§^ *p* < 0.05, ^§§^ *p* < 0.01, ^§§§^ *p* < 0.001 vs. STZ; ^##^ *p* < 0.01, ^#^ *p* < 0.05 vs. STZ + LG1; °° *p* < 0.01, ° *p* < 0.05 vs. STZ + LG2. *n* = 3 in all graphs.

Nuclear factor erythroid 2-related factor 2 (Nrf2) acts as a redox-sensitive transcription factor whose activity, elicited by increases in free radicals, regulates multiple genes encoding anti-oxidant enzymes, including, among others, NADPH dehydrogenase quinone oxido-reductase 1 (NQO1) [30]. As shown in Figure 6A, Nrf2 protein levels were considerably increased in the retinas of the STZ and the STZ + Lipo groups compared to the controls. The treatments with either LG1 or LG2 were not effective since, in both the STZ + LG1 and the STZ + LG2 groups, the Nrf2 levels were not significantly different from those of the STZ mice. In contrast, both doses of LipoLG seemed to completely abolish the STZ-induced increase in Nrf2. In particular, Nrf2 expression was significantly decreased in the STZ + LipoLG1 compared to the STZ + LG1 group and in the STZ + LipoLG2 compared to the STZ + LG2 group. Consistent with the effect of LipoLG1 and LipoLG2 on Nrf2 retinal content, the NQO1 levels in the STZ + LipoLG1 and the STZ + LipoLG2 groups also significantly reduced compared to those of the STZ group and were similar to those in the control retinas. In contrast to the Nrf2 data, NQO1 levels decreased compared to those in the STZ animals, as well as in the STZ + LG1 and STZ + LG2 groups. Overall, these observations of oxidative stress markers indicated the protective effects of both LG and LipoLG at the lowest dose employed in the experiments; however, the effects of LG alone on the regulation of NQO1 could be appreciated but the same was not true for that of Nrf2.

NF-kB is an oxidant-sensitive transcription factor responsible for regulating the gene expressions of the factors involved in inflammatory responses [31]. As shown in Figure 6C, NF-kB phosphorylation was significantly increased in the retinas of the STZ group, and no changes were detected in the STZ + Lipo, STZ + LG1, and STZ + LG2 groups. In contrast, both doses of LipoLG were effective in preventing this increase, with significant effects with respect to the relative doses of LG. Regarding inflammation, the glial fibrillary acidic protein (GFAP) is a marker of astrogliosis, a phenomenon that is typically observed in proliferative retinopathies such as DR [32]. GFAP levels were significantly increased in the retinas of the STZ and STZ + Lipo groups. In the groups treated with LG, a decreasing trend for GFAP levels could be observed, but no statistically significant differences were observed with respect to the STZ group. Complete protection from pathologic increases of GFAP was found in the STZ + LipoLG1 and in the STZ + LipoLG2 groups. Overall, the data relative to the inflammation markers indicated efficient protection conferred by both doses of LipoLG, while only limited effects on reactive gliosis could be detected with the LG treatments.

**Figure 7:**
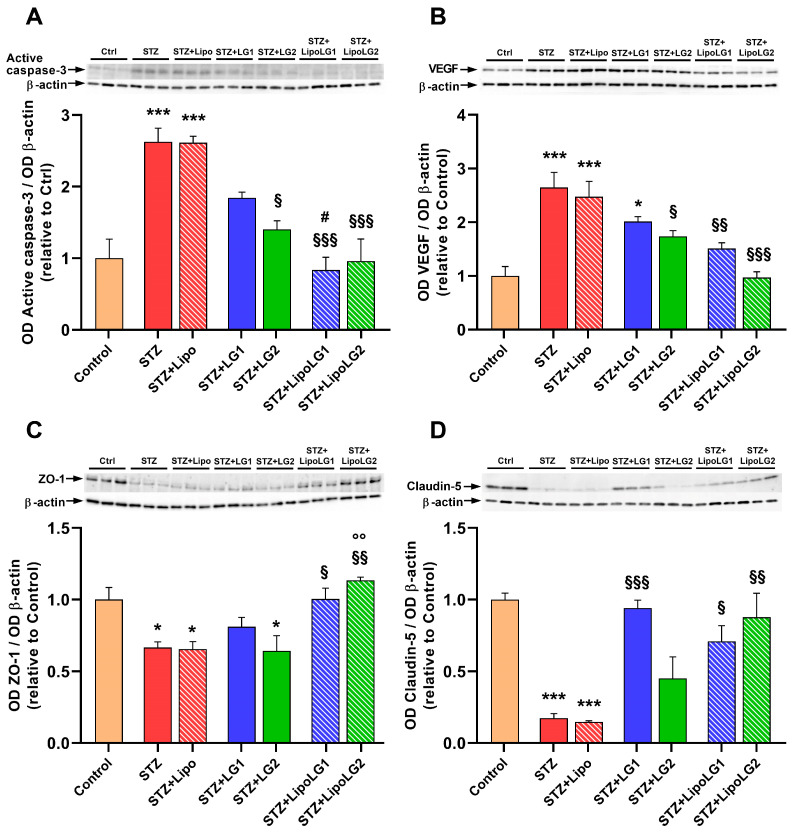
Western blot analysis showing representative immunoreactive bands and quantitative densitometric analysis of the protein levels of active caspase-3 (**A**), VEGF (**B**), ZO-1 (**C**), and Claudin-5 (**D**) in the different experimental groups. * *p* < 0.05, *** *p* < 0.001 vs. control; ^§^ *p* < 0.05, ^§§^ *p* < 0.01, ^§§§^ *p* < 0.001 vs. STZ; ^#^ *p* < 0.05 vs. STZ + LG1; °° *p* < 0.01 vs. STZ + LG2. *n* = 3 in all graphs.

Apoptotic cell death and VEGF upregulation are two of the main hallmarks of DR [14]. Apoptosis was investigated by measuring the levels of the activated form of caspase-3 (Figure 7A). As expected, the levels of this marker were significantly increased in the retinas of both the STZ and the STZ + Lipo groups. The treatment with LG was effective in decreasing active caspase-3 levels. However, a statistically significant effect was observed only with the lowest dose of LG (STZ + LG2 group). In contrast, both doses of LipoLG (STZ + LipoLG1 and STZ + LipoLG2 groups) completely prevented any increases in the apoptotic markers. The levels of active caspase-3 in the STZ + LipoLG1 group were significantly lower than those in the STZ + LG1 group. A similar pattern of responses was observed in the levels of VEGF (Figure 7B). The increase in VEGF protein observed in the STZ and in the STZ + Lipo groups was contrasted by the LG treatments, although a statistically significant effect was observed only in the STZ + LG2 group. The pathologic VEGF increase was completely prevented by either treatment involving LipoLG (STZ + LipoLG1 and STZ + LipoLG2 groups). Overall, these data indicate that LG is protective against apoptosis and VEGF upregulation; however, stable and complete protection is only achieved with LipoLG treatments. In addition, both doses of LipoLG appeared to have comparable efficacy.

Zonula occludens-1 (ZO-1) and claudin-5 are tight junction proteins of the BRB [33]. The levels of both of them were significantly reduced in the retinas of both the STZ and the STZ + Lipo groups (Figure 7C,D). The treatment with the highest LG dose (STZ + LG1 group) led to an increase in ZO-1 protein levels, although this effect was not significantly different from those in the STZ group, while the lowest LG dose (STZ + LG2 group) was not effective. In contrast, both doses of LipoLG (STZ + LipoLG1 and STZ + LipoLG2 groups) prevented the STZ-induced decrease in ZO-1 levels (Figure 7C). In particular, ZO-1 expression was significantly increased in the STZ + LipoLG2 compared to the STZ + LG2 group. A similar pattern was observed for the claudin-5 levels, with the only difference being that the treatment with LG1, in this case, appeared to induce a statistically significant recovery of the protein levels (Figure 7D). Overall, these data regarding BRB markers indicated the powerful protective effects of both doses of LipoLG in the presence of diabetes-induced BRB damage, while the effects of LG alone were weaker.

## 4. Discussion

The present work confirms and expands on the evidence of the powerful, protective effects of LG administration against retinal disease that we have reported in previous papers [20,22]. The fact that the mice could drink water, LG, or LipoLG with no apparent preference and with no evidence of side effects over an extended period of time indicates that a LG-based therapy to treat retinal diseases may be well tolerated, which is a fundamental aspect in view of possible applications in clinics. Most importantly, the present study’s results show the enhanced effects of LG-encapsulated liposomes, which prevent DR-induced functional retinal failure, oxidative stress, inflammation, astrogliosis, cell apoptosis, VEGF upregulation, and BRB breakdown, indicating that the therapeutic potential of LG can be greatly improved by the use of nanocarriers that may increase LG bioavailability.

Nutraceuticals have been reported to be anti-inflammatory, anti-oxidant, anti-cancer, and neuroprotective molecules that may play positive roles in a variety of diseases [34]. However, their actual efficacy is much lower than expected. Indeed, these compounds, due to their intrinsic physicochemical characteristics, are affected by rapid chemical degradation, low solubility, and poor bioavailability after oral ingestion due to gastric pH, enzyme action, and intestinal microbiota [35,36]. A large number of natural foods (or their derivatives) with potential therapeutic applications have been identified in recent years, and improving their bioavailability is far more important than identifying new bioactive compounds.

A PubMed search using “nutraceutical & bioavailability” as search terms retrieved 94 review papers published during the first eight months of 2023 in which different systems for improved nutraceutical delivery were considered. They include the use of lipid or glass nanoparticles, dendrimers, nanocapsules, micelles, lipid nanocarriers, nanoemulsions, scaffolds, hydrogel nanocomposite, or nanocages [37,38,39]. In addition, a variety of nanoparticles made of organic compounds like carbohydrates, proteins, fats, or inorganics such as oxides of silver and titanium are also available for nutraceutical delivery [24]. Other methods may include the use of starch, which is an abundant, low-cost, edible, and biodegradable native polymer that has been recently investigated as a potential base for new delivery systems [40]. Similarly, plant proteins may represent green, alternative nanocarriers thanks to their amphiphilic nature, which makes them compatible with many bioactive compounds to enhance their solubility, stability, and bioavailability [41]. Another recently considered nanocarrier is pectin, a polysaccharide that is easily extracted from natural sources and is biodegradable, biocompatible, and non-toxic. Together with proteins, it may form nanostructures that facilitate nutraceutical passage through the gastrointestinal system and absorption [42]. This brief survey of the current literature demonstrates the existence of ongoing and variegated research aimed at improving the efficacy of nutraceuticals as therapeutic treatments in different diseases. Some efforts have also been dedicated to increasing the bioavailability of nutraceuticals in the context of retinal diseases, but regarding this topic, the available literature only contains some investigations using different types of nanoparticles to improve the efficacy of curcumin, resveratrol, and epigallocatechin gallate for the treatment of DR (see [1] for a review).

Among nanocarriers, liposomes have been widely investigated and demonstrated to be particularly efficient in protecting the encapsulated compound from physiological degradation and extending its half-life. Thanks also to their excellent biocompatibility and safety, drug delivery through liposomes is associated with superior therapeutic effects and fewer toxic side effects. Several liposome-based drugs have been approved by both the U.S. Food and Drug Administration and the European Medicines Agency [43]. Liposome-mediated drug delivery to the retina has been associated with intravitreal administration or topical applications [44,45]. To our knowledge, the present work is the first to document liposome-mediated oral delivery of a nutraceutical to treat a retinal disease, and the highly positive results of this study indicate that this approach warrants further development.

In our studies, the ERG data demonstrated that treatments with LG or with LipoLG can rescue retinal function in diabetic mice. In particular, the photoreceptor activity (represented by the scERG responses [46]), the bipolar cell responses (whose activity is included in the scERG b-wave [46]), the overall synaptic activity in the inner plexiform layer (recorded in the OPs [47]), and the activity of RGCs (represented in the PhNR and PERG amplitudes [48]) were significantly ameliorated in the experimental groups treated with LG or with LipoLG. It must be noted, however, that the treatment with LG was associated with significant functional improvements only when a high dose was administered, while a low dose of LG failed to induce any effects in any of the ERG experiments. In addition, with the exception of the PhNR data, both the high and the low dose of LipoLG displayed comparable efficacy, indicating that its encapsulation in liposomes allows LG to display full protective capacity at one-tenth of the concentration at which the free LG shows protective effects. The low dose of LipoLG did not exert any effects upon the PhNR responses, though it rescued the PERG amplitudes, two different parameters linked to RGC activity. Since PERG is considered a more reliable indicator of RGC function than PhNR [48,49], we favor the possibility that LipoLG2 is indeed efficient in rescuing RGC responses.

As expected, the protective effects of LG or LipoLG on retinal function were concomitant with the protective effects of LG or LipoLG on retinal molecular markers typical of DR. Overall, the effects of LG described here are similar to those reported in a previous study of diabetic rat retinas in which LG was not spontaneously assumed by the animals but was administered daily via gavage [20]. However, some discrepancies were also noted, including the poorly defined effect on oxidative stress (LG induced clear downregulation of NQO1 but not of Nrf2 in retinas of diabetic mice) and the lack of effects of LG on inflammation markers such as the pNF-kB/NF-kB ratio, although protective effects against astrogliosis were observed. In contrast to LG, both doses of LipoLG were highly effective in preventing changes related to the diabetic condition in all of the molecular markers examined in these studies. The clear advantage represented by the use of LipoLG over LG is likely to be due to the improved bioavailability of the compound. The fact that, unlike the LG effects, those of LipoLG did not exhibit dose dependency indicates that the lowest dose of LipoLG tested in these investigations was sufficient to confer maximal protective effects. Dedicated studies with an extended dose–response evaluation will be performed to determine the lowest effective dose of freely assumed LipoLG in models of DR. In addition, in view of the recently reported protective role of LG on RGCs in glaucoma [22], it will be interesting to test possible improvements in the therapy using LipoLG in glaucoma models.

## 5. Conclusions

This study demonstrates that the efficacy of LG in reducing or preventing the manifestation of DR in diabetic animals is greatly enhanced by LG encapsulation in liposomes. An aqueous preparation of LipoLG was spontaneously assumed by the experimental animals and was not associated with apparent side effects, indicating good tolerance towards the treatment. These observations may represent the basis for the development of dietary supplements that, perhaps when combined with other therapies, could determine a fundamental step forward not only for the treatment of DR but also for its prevention.

## Figures and Tables

**Table 1:** Antibodies used for Western blotting.

Antibodies *	Dilution	Source	Catalog n.
Rabbit monoclonal anti-Nrf2	1:1000	Abcam, Cambridge, UK	ab92946
Rabbit polyclonal anti-NQO1	1:500	Abcam	ab34173
Rabbit polyclonal anti-pNF-kB p65	1:100	Santa Cruz Biotechnology, Dallas, TX, USA	sc-33020
Rabbit polyclonal anti-NF-kB p65	1:1000	Abcam	ab16502
Rabbit monoclonal anti-GFAP	1:500	Abcam	sab1301592
Rabbit monoclonal anti-cleaved caspase-3	1:500	Cell Signaling Technology, Danvers, MA, USA	9664
Rabbit polyclonal anti-VEGF	1:1000	Abcam	ab9570
Rabbit polyclonal anti-ZO-1	1:500	Invitrogen—Thermo Fisher Scientific, Waltham, MA, USA	40-2200
Mouse monoclonal anti-Claudin-5	1:500	Invitrogen—Thermo Fisher Scientific (Waltham, MA, USA)	35-2500
Mouse monoclonal anti-β-actin	1:2500	Sigma Aldrich-Merck, St. Louis, MO, USA	a2228
Goat polyclonal IgG HRP-conjugated anti-rabbit	1:5000	Bio-Rad Laboratories, Inc., Hercules, CA, USA	1706515
Rabbit polyclonal IgG HRP-conjugated anti-mouse	1:5000	Sigma-Aldrich-Merck	A9044

* Abbreviations: GFAP, Glial fibrillary acidic protein; HRP, horseradish peroxidase; NF-kB p65, p65 subunit of nuclear factor kappa-light-chain-enhancer of activated B cells; NQO1, NADPH dehydrogenase quinone oxido-reductase 1; Nrf2, nuclear factor erythroid 2-related factor 2; pNF-kB p65, NF-kB p65 phosphorylated at Ser 276. VEGF, vascular endothelial growth factor; ZO-1, Zonula occludens-1.

**Table 2:** Animal treatments and experimental groups.

Treatment	Experimental Groups						
	Control	STZ	STZ + Lipo	STZ + LG1	STZ + LG2	STZ + LipoLG1	STZ + LipoLG2
Citrate buffer (i.p.)	✓						
STZ (i.p.)		✓	✓	✓	✓	✓	✓
Water	✓	✓					
Lipo			✓				
LG 1:10				✓			
LG 1:100					✓		
LipoLG 1:10						✓	
LipoLG 1:100							✓

## Data Availability

The data presented in this study are available on request from the corresponding authors.
